# Function Over Mass: A Meta-Analysis on the Importance of Skeletal Muscle Quality in COVID-19 Patients

**DOI:** 10.3389/fnut.2022.837719

**Published:** 2022-04-20

**Authors:** Flaydson Clayton Silva Pinto, Márcia Fábia Andrade, Guilherme Henrique Gatti da Silva, Jaline Zandonato Faiad, Ana Paula Noronha Barrére, Renata de Castro Gonçalves, Gabriela Salim de Castro, Marília Seelaender

**Affiliations:** ^1^Cancer Metabolism Research Group, Department of Surgery, LIM26 HC-USP, University of São Paulo, São Paulo, Brazil; ^2^Departamento de Biologia Celular e do Desenvolvimento, Instituto de Ciências Biomédicas, Universidade de São Paulo, São Paulo, Brazil; ^3^Biology Department, Brandeis University, Waltham, MA, United States

**Keywords:** COVID-19, sarcopenia, SARS-CoV-2, coronavirus, disease outcome, skeletal muscle, muscle density, skeletal muscle index

## Abstract

COVID-19 caused by SARS-CoV-2 infection is a highly contagious disease affecting both the higher and lower portions of the respiratory tract. This disease reached over 265 million people and has been responsible for over 5.25 million deaths worldwide. Skeletal muscle quality and total mass seem to be predictive of COVID-19 outcome. This systematic review aimed at providing a critical analysis of the studies published so far reporting on skeletal muscle mass in patients with COVID-19, with the intent of examining the eventual association between muscle status and disease severity. A meta-analysis was performed to evaluate whether skeletal muscle quantity, quality and function were related to disease severity. Systematic reviews and meta-analyses were conducted according to the guidelines of the Cochrane Handbook for Systematic Reviews of Interventions and reported according to the guidelines of the PRISMA (Preferred Reporting Items for Systematic Reviews and Meta-Analysis) guide. From a total of 1,056 references found, 480 were selected after removing duplicates. Finally, only 7 met the specified inclusion criteria. The results of this meta-analysis showed that skeletal muscle quality, rather than quantity, was associated with COVID-19 severity, as confirmed by lower skeletal muscle density and lower handgrip strength in patients with severe disease. Muscle function assessment can thus be a valuable tool with prognostic value in COVID-19.

## Introduction

COVID-19 a disease caused by the SARS-CoV-2 virus infection, is highly contagious, affecting both the higher and lower portions of the respiratory tract. This disease has been responsible for 5.25 million deaths worldwide so far (1). COVID-19 begins with typical viral infection-induced symptoms such as cough, fever, dyspnea, and myalgia, accompanied by leukopenia, but some patients progress to bilateral respiratory distress and increased susceptibility to secondary infections (2). In the severe cases, abnormal coagulation, excessive inflammation, lower oxygen saturation and oxidative stress are observed, leading to kidney and liver failure and death (2). The most critical determinant of disease severity is age, with individuals over 65 years of age having the most significant risk for intensive care requirement (3). The age-associated increase in inflammation is paralleled by chronic augment of circulating inflammatory biomarkers such as interleukin-6 (IL-6), tumor necrosis factor-alpha (TNF-α) and C-reactive protein (CRP) (4). Other risk factors were identified in severe and critical COVID-19, including comorbidities such as hypertension, diabetes, obesity, pulmonary and cardiovascular disease (5). Sarcopenia, loss of skeletal muscle mass and function, contributes to high morbidity and mortality in the elderly population (3). In addition, sarcopenic patients also show higher levels of circulating CRP (6), along poor immune response and metabolic stress, when facing acute infection, major surgery, and other stressor stimuli (7). Various studies report that sarcopenia is associated with longer hospital stay, increased frequency of employment of mechanical ventilation, and increased mortality, in ICU patients (8–11).

Sarcopenia has been defined by the European Working Group on Sarcopenia in Older People (EWGSOP2) as low muscle strength, low muscle quantity or quality, and in severe cases, low physical performance is also present (12). Therefore, it is important to distinguish between different nomenclature: while “myopenia” refers only to low muscle mass; “myosteatosis” indicates intramuscular lipid infiltration and negatively impacts muscle quality and also, patient mobility, increasing frailty (13–15). Both can happen independently, despite having a possible synergic action and age plays a role in aggravating this scenario (15). Sarcopenia may result from a combination of these deleterious alterations, or still, by other muscle morphology and function disruptive modifications, such as loss of innervation, among other (16, 17).

This correlation between body composition and COVID-19 was investigated in a study where patients with lower vastus lateralis cross-sectional muscle area were also those with higher length of hospital stay when compared with patients in the mid/highest tertiles (18). Furthermore, lower handgrip strength was similarly associated with increased hospital stay (18). Patients who died of COVID-19 also presented lower pectoralis muscle density, as measured in Hounsfield units (HU) by computerized tomography (CT), than did survivors (19). Finally, lipid infiltration in muscles at the level of the twelfth thoracic vertebra was positively correlated with mortality in patients with COVID-19 (20). Thus, muscle mass quantity and quality may be associated to prognosis in patients hospitalized due to SARS-COV-2 infection. This systematic review aimed to critically analyze the studies published so far reporting on skeletal muscle mass in patients with COVID-19, and intended to detect possible associations between muscularity and disease outcome.

## Methods

Systematic reviews and meta-analyses were performed according to the guidelines of the Cochrane Handbook for Systematic Reviews of Interventions (21) and reported according to the guidelines of the PRISMA (Preferred Reporting Items for Systematic Reviews and Meta-Analysis) (22) guide.

### Literature Search Strategy

A literature search of observational studies was performed to investigate whether skeletal muscle quantity, quality and function were related to disease severity in patients with COVID-19, searching 3 literature databases. With the help of the search string, a researcher (JZF) searched the database (last search date in November 2021) of the Web of Science, PubMed, and LILACS. Not any restrictions were applied to the initial electronic search. For retrieval of studies, the following MeSH terms were used: “SARS-CoV-2” OR “COVID-19” OR “coronavirus” AND “muscle mass” OR “muscle strength” OR “skeletal muscle” OR “sarcopenia.”

### Eligibility Criteria

After removing duplicates and irrelevant material, the titles and abstracts identified in the search were independently selected by 4 investigators (APNB, GSC, JZF, MFA). Potentially eligible studies were analyzed by 3 investigators (APNB, GSC and MFA). Disagreements among reviewers were discussed by MFA, JZF and RCG and decided by consensus, involving all authors.

The selected studies met the inclusion requirements on the patient, intervention, comparison, and outcome (PICO) strategy as presented in Table 1. Studies evaluating body composition in patients with confirmed, active infection by SARS-CoV-2; evaluation of muscle quality, quantity, and strength assessed by computerized tomography (CT) or similar method, employment of dynamometer (grip strength); only fully published studies. The exclusion criteria included: studies that lacked RT-PCR positive patients for COVID-19; studies that did not assess the association between skeletal muscle and/or muscle function and risk of COVID-19 severity; studies employing experimental models and/or *in vitro* analyses; studies with pediatric patients; studies in languages other than English; case report studies; procedural studies; academic papers; literature reviews; cards; and studies missing skeletal muscle assessment data.

**Table 1:** Inclusion and exclusion criteria performed by patient, intervention, comparison, and outcome (PICOS) strategy.

	**Inclusion criteria**
Population	Patients > 18 years old
Intervention/Exposure	Patients with severe COVID-19 disease confirmed by a positive SARS-COV-2 test
Counterpart	Non-severe COVID-19 confirmed by a positive SARS-COV-2 test
Outcome	Studies that evaluated muscle quantity and quality by CT or similar methods
Study design	Observational studies

### Extraction and Synthesis of Data

Independent data extraction was performed in duplicate by all the authors using pre-designated data collection forms, crossing differences, and making corrections where appropriate was done. The data extracted from each study were as follow: (a) general information about the selected study (i.e., author, journal, and year of publication); (b) information on the intervention category and control counterparts; (c) population included in the study, information on the analysis of parameters and overall effect size analysis; (d) primary results related to the purpose of the systematic review; (e) methods of evaluating the association between the studied outcomes, and (f) discussion.

### Assessing the Quality of Trials

All the investigators assessed the quality of evidence using The Newcastle-Ottawa Scale (NOS) of the included cross-sectional studies, retrospective and prospective cohort studies, based on the studies' patient selection, adjustment for potential confounding variables, and outcome assessment (23). This scale assesses a maximum of 9 points assigned to each study and articles with a NOS score >5 were considered as of high quality (23).

### Statistical Analysis of Data

Meta-analysis was performed on the extracted data, where applicable, using a random-effects model in Review Manager version 5.4.1 (RevMan) (24). Initially, data were organized and standardized by two investigators (FCSP and RCG) to facilitate the analysis. From those studies with multiple time points, only the final was included in the overall meta-analysis. Data extracted were standardized to obtain mean and standard deviations (SD) for analysis. The authors were contacted to clarify any doubts about the articles. Unpublished data were requested, however, only the assessment of number of deaths caused by COVID-19 was used to perform the analysis (18).

When reported, data were then presented as the median and interquartile range (IQR) following conversion adopting a specific formula (25), while SDs were obtained according to the Cochrane Handbook (21). In addition, individual analysis was performed to explore the effect of each skeletal muscle assessment method (skeletal muscle density and index, and grip strength) on overall outcome. Finally, the mean difference (MD) between counterparts with 95 % CIs was adopted to express the absolute difference between the mean values.

Finally, the heterogeneity of results among the studies was determined by *I*^2^, where ≤ 49.9 % were considered low values, 50–74.9 % medium and 75–100 % indicative of high heterogeneity. The z-score was employed as general effect test, considering *p* ≤ 0.05 as significant as recommended by the Cochrane Handbook (21).

## Results

### Study Identification and Selection

**Figure 1:**
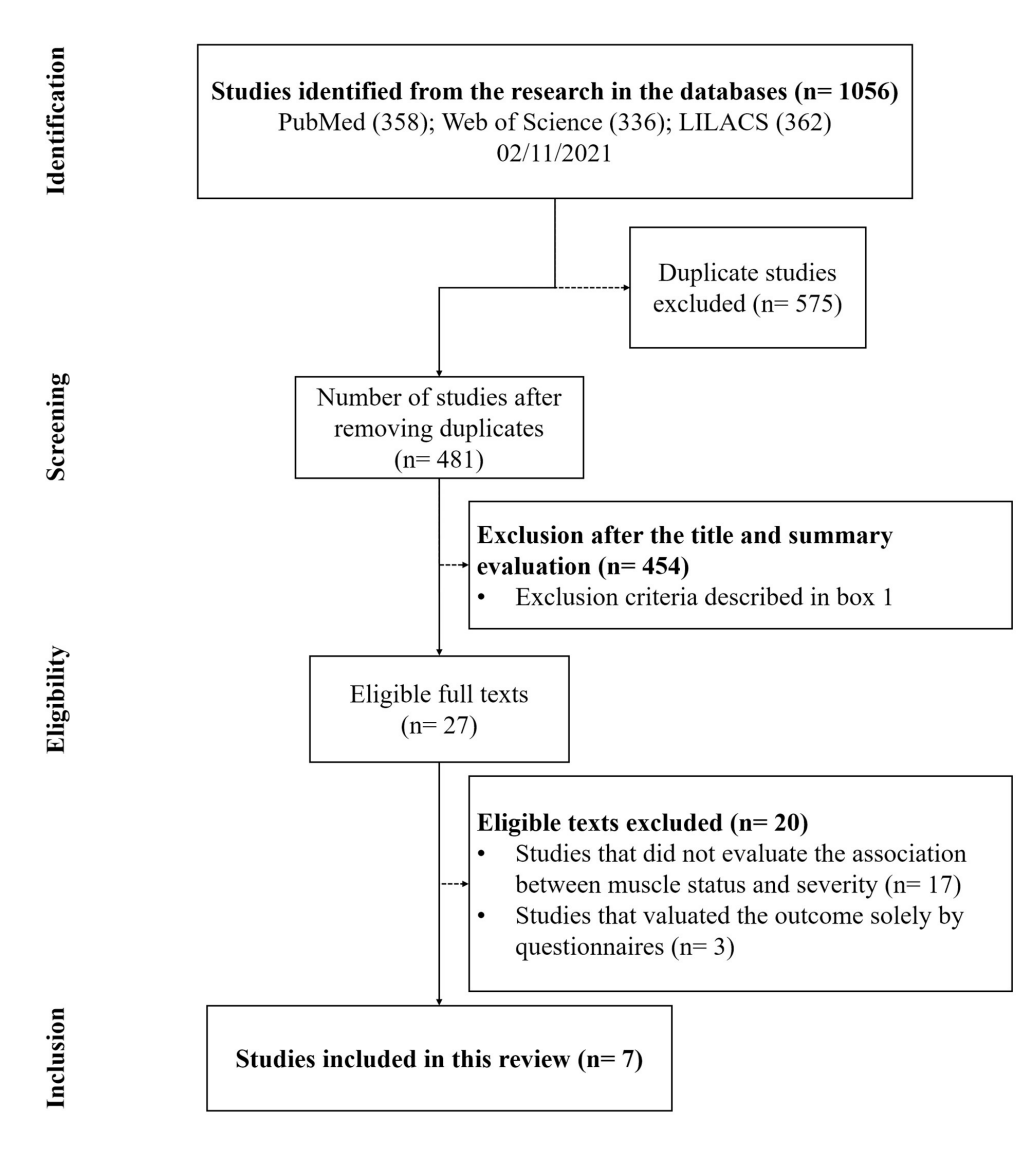
The PRISMA flowchart of systematic process.

In total, 1,056 references were found. After removing the duplicates (575), 481 articles remained. From those 129 review articles, 18 editorials, 20 in languages other than English, and 287 studies were excluded, after reading the title and abstracts, as they failed to match the inclusion criteria (Box 1). After reading the full texts, 21 studies were removed for not addressing the association between skeletal muscle status and COVID-19 infection severity (17) or for having evaluated outcomes exclusively by questionnaires; or for assessing pediatric populations. Seven (18, 20, 26–30) studies thus remained and were included in this systematic review (Figure 1) (22). The main characteristics of these selected studies are described in Table 2.

Box 1 **Reasons for exclusion of studies.**

**Table:** 

**Exclusion criteria**	***n***
Did not assess body composition	71
Did not assess COVID-19 positive RT-PCR patients	147
Assessed post-COVID-19 patients	32
Animal model	10
Review	129
Other language	20
Editorial material	18
Letter	15
Proceedings papers	2
Unavailable paper	2
Meeting abstract	8

**Table 2:** Main characteristics of the studies included in the meta-analysis.

**Study**	**Subjects M/F^a^**	**Age (years)^b^**	**Type of study**	**Sample size**	**Death cases**	**Length of stay (days)**	**Main outcomes**	**Other outcomes**
Tuzun et al. (28)	Hospitalized COVID-19 patients All: 77/73 Severe: 25 /22 Non-severe: 52/51	All: 53.17 ± 15.49 Severe: 58.81 ± 14.50 Non-severe: 50.60 ± 15.31	Cross-sectional Study	All: 150 Severe: 47 Non-severe: 103	NR	NR	Lower grip strengths in Female patients with severe infections.	CRP was significantly higher in Lower grip strengths, female patients.
Kara et al. (26)	Hospitalized COVID-19 patients All: 172/140 Mild: 65/50 Moderate: 86/73 Severe: 21/17	All: 46.1 ± 14.8 Mild: 39 (21–74)^c^ Moderate: 46 (20–90)^c^ Severe: 61 (42–90)^c^	Cross-sectional Study	All: 312 Mild:115 Moderate: 159 Severe: 38	NR	All: 9 (2–30)^c^ Mild: 8 (2–19)^c^ Moderate: 8 (3–21)^c^ Severe: 18 (6–30)^c^	Length of hospital stay and CRP were higher in the severe group vs. other groups. In addition, mean grip strength values were lower in severe vs. other groups.	BMI was lower in the mild vs. other groups. Age, obesity, CRP level, and low grip strength were found to be independent predictors for severe disease.
Rossi et al. (27)	Severe COVID-19 patients admitted in ICU All: 121/32 Survivors: 100/26 Deaths: 21/6	All: 64.19 ± 9.98 Survivors: 63.32 ± 10.50 Deaths: 68.26 ± 5.55	Cohort Prospective Study	All: 153 Survivors: 126 Deaths: 27	27	NR	Survivors showed lower age, BMI, IMAT area, and CRP than death subjects.	CRP level was significantly higher in subjects in the highest IMAT/muscle tertile than subjects in the lowest tertile.
Viddeleer et al. (20)	Hospitalized COVID-19 patients All: 129/86 Alive: 102/73 Dead: 27/13	All: 61.1 ± 14.3 Alive: 59.8 ± 14.5 Dead: 66.9 ± 12.0	Cohort Prospective	All: 215 Alive: 175 Dead: 40	40	NR	Non-survivors had a larger CSA of IMAT and a more extensive IMAT index compared with survivors.	Patients who died were older and more frequently invasively ventilated.
Gil et al. (18)	Hospitalized COVID-19 patients All: 93/93 Survivors: 86/88 Deaths: NR	All: 59 ± 15 Survivors: NR Deaths: NR	Cohort Prospective Study	All: 186 Survivors: 174 Deaths: 12	12	All: 7 (4–11)^d^ Survivors: 7 (4–11)^d^ Deaths: NR	Muscle strength and mass (vastus lateralis by US) are predictors of LOS in patients with moderate to severe COVID-19.	An association between increased handgrip strength and shorter hospital stay was identified when standardized handgrip strength. The mean LOS was shorter for the most muscular patients vs. others. The mean LOS for the patients with the lowest CSA was longer.
Yi et al. (30)	Hospitalized COVID-19 patients All: 133/101 Severe: 23/8 Non-severe: 110/93	All: 44.5 (2.0–81.0)^c^ Severe: 45.0 (26.0–80.0)^c^ Non-severe:43.0 (2.0–81.0)^c^	Cohort Retrospective Study	All: 234 Severe: 31 Non-severe: 203	NR	NR	Myosteatosis seems to be associated with a higher risk of transition to severe illness in patients affected by COVID-19 who initially presented mild infection.	Patients with severe illness showed significantly higher SMFI and higher incidence of myosteatosis.
Yang et al. (29)	Hospitalized COVID-19 patients All: 70/73 Critical: 27/18 Non-critical: 43/55	All: 66 (56–73.5)^c^ Critical: 67 (60–75)^c^ Non-critical: 65 (54.3–73)^c^	Cohort Retrospective Study	All: 143 Critical: 45 Non-critical: 98	Critical: 15 Non-critical: 0	NR	Patients with VA or high IMF deposition were older, and they had significantly higher risks for MV than patients without those features. Furthermore, VA or high IMF deposition were independent risk factors for critical illness.	Patients aged <60 years with visceral adiposity and high IMF deposition had higher risks for critical illness.

a *M/F, male/female*.

b *Completed patients.Values are mean ± SD unless otherwise specified*.

c *Median (min to max)*.

d *Median (IQR)*.

*NR, not reported data; CRP, C-reactive protein; BMI, Body mass index; CSA, Cross-sectional area; IMAT, Intermuscular adipose tissue; SMFI, Septocutaneous muscle fat index; MFI, Muscle fat index; VA, visceral Adiposity; IMF, Intramuscular fat; MV, Mechanic ventilation*.

### Study Characteristics

All studies employed the real-time polymerase chain reaction (RT-PCR) test to confirm SARS-CoV-2 infection. Of the 7 studies included in the analysis, 2 were carried out in Turkey (26, 28), 2 in China (29, 30), 1 in Italy (27), 1 in the Netherlands (20) and 1 in Brazil (18). The sample size of the studies ranged from 100 to 234 patients.

To perform this meta-analysis, the patients in the studies were classified presenting or not severe disease. Severe groups included patients who died as a consequence of COVID-19 or recovered, after presenting severe disease, while the non-severe group included the survivors who presented the mild and non-critical forms of COVID-19, according to the classification adopted in each article. Three studies classified severe disease as the presence of pneumonia (fever, cough, dyspnea and tachypnea), along with decreased blood oxygen content (below 90%) and extensive lung involvement, as detected by CT (i.e., CT score >11) or CT findings (bilateral multifocal ground-glass opacities ≥50%) compatible with the disease. Mild illness was implied that lung images were comparable to standard chest CT and/or radiographic findings (26, 27, 30). Tuzun et al. (28) defined COVID-19 severity according to the American Thoracic Society (ATS) guidelines for community-acquired pneumonia. Yi et al. (30) determined illness severity according to the Chinese Management Guideline for COVID-19. Acute respiratory distress syndrome (ARDS) and sepsis were used to classify critically ill patients in Yang et al. (29). Rossi et al. (27), Viddeleer et al. (20), and Gil et al. (18) employed death caused by COVID-19 to stratify disease severity.

### Quality Assessing

**Figure 2:**
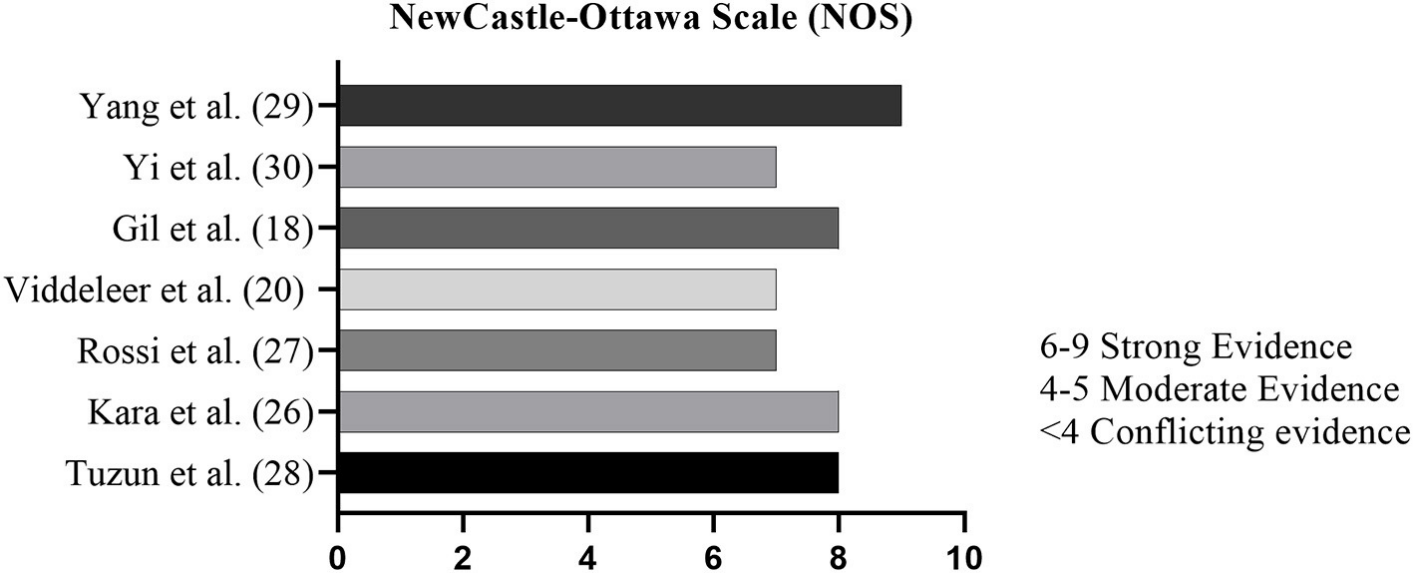
The Newcastle-Ottawa Scale (NOS).

Studies were assessed for methodological quality using the NOS (23). Selection, Comparability and Outcome are evaluated in 8 items of this scale (23). For each item, one point is credited to the study, except for “Cohort comparability based on design or analysis,” which can score twice, reaching a maximum score of 9 points. Studies that are comprised of strong evidence are those presenting scores from 6 to 9. Moderate quality studies are considered those that score 4–5 out of 9 possible points. Studies scoring lower than 4 points are regarded as of limited evidence. According to the results of NOS assessment, a low risk of bias was observed in the “selection” category, as well as “comparability” and “outcome” measured (Figure 2). Some studies proved to be unrepresentative (20, 26, 27), as most recruited patients were critically ill ICU patients, coming from a highly heterogeneous population of patients with positive results for COVID-19. Data represented in the studies, in general, proved to be reliable, having been mainly obtained from patients' hospital records. Some studies did not achieve minimum score in the item “Follow-up was sufficient for the outcome to occur” due to the short study follow-up time (20, 30).

### Body Composition and Risk of Complications During SARS-CoV-2 Infection

#### Skeletal Muscle Index

**Figure 3:**
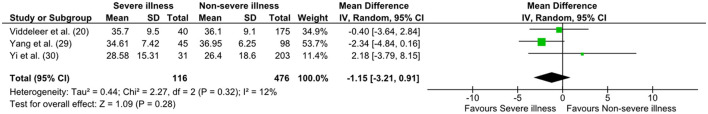
The forest plot of SMI and survival rate. SD, standard deviation; CI, confidence intervals.

Two studies reported SMI (20, 30), and another one (29) described skeletal muscle area and height, providing the data for SMI calculation. Thus, a total of 592 patients presented data regarding this parameter (Table 3). Yi et al. (30) and Yang et al. (29) CT analyses explored T12, associating COVID-19 severity with body composition (Severe 28.58 cm^2^/m^2^ ± 15.31 and 34.61 cm^2^/m^2^ ± 7.42; non-severe 26.4 cm^2^/m^2^ ± 18.6 and 36.95 cm^2^/m^2^ ± 6.25, respectively). Viddeleer et al. (20) CT analysis was based on images at L3 level (severe 35.7 ± 9.5; non-severe 36.1 ± 9.1) and assessed the association between COVID-19 survival and body composition. The overall effect did not show statistical significance (*p* = 0.28), despite the low heterogeneity (*I*^2^ = 12 %) [MD = 1.15; 95% CI: −3.21, −0.91; *Z* = 1.09; *p* = 0.28 (Figure 3)].

**Table 3:** Baseline patient's data from all included studies in this meta-analysis.

**Study**	**Initial BMI (kg/m^**2**^)**	**Handgrip strength (kg/f)**	**Muscle cross-section area (cm^**2**^)**	**Muscle Density (HU)**	**SMI (cm^**2**^/m^**2**^)**
Tuzun et al. (28)	NR	Non-severe: 30.59 ± 13.39 Severe: 27.38 ± 14.04	NR	NR	NR
Kara et al. (26)	Mild: 26.8 ± 5.3 Moderate: 29.3 ± 5.4 Severe: 30.5 ± 6.6	Mild: 35.1 ± 11.2 Moderate: 34.7 ± 11.1 Severe: 26.5 ± 12.4	NR	NR	NR
Rossi et al. (27)	All: 29.30 ± 4.58 Survivors: 28.25 ± 4.43 Deaths: 30.58 ± 5.29	NR	All: 16.66 ± 9.37 Survivors: 16.46 ± 9.40 Deaths: 17.59 ± 9.34	All: 37.79 ± 8.55 Survivors: 38.38 ± 8.58 Deaths: 35.05 ± 8.03	NR
Viddeleer et al. (20)	All: 28.9 ± 6.1 Alive: 28.8 ± 6.2 Dead: 29.0 ± 5.8	NR	All: NR Alive: 108.0 (86.5–124.4)^a^ Dead: 104.0 (83.3–116.7)^a^	All: NR Alive: 27.6 ± 10.9 Dead: 24.0 ± 10.1	All: NR Alive: 36.1 ± 9.1 Dead: 35.7 ± 9.5
Gil et al. (18)	All: 29.5 ± 6.9	All: 21 (15–30)^a^ Survivors: 22 (15–30)^a^ Deaths: ^*^	All: 12 (12–19)^a^^#^ Survivors: 16 (12–18)^a^^#^ Deaths^*^	NR	NR
Yi et al. (30)	NR	NR	NR	NR	All: 24.2 (15.3–40.2)^b^ Severe:25.4 (20.5–40.2)^b^ Non-severe: 23.7 (15.3–37.3)^b^
Yang et al. (29)	All: 23.4 (21.9–25.3)^a^ Critical: 24.8 (22.5–26.1)^a^ Non-critical: 23 (21.4–24.8)^a^	NR	All: 96.2 (79.0–118.2)^a^ Critical: 93.3 (77–118.4)^a^ Non-critical: 98.5 (81.7–117.2)^a^	All: 32.3 (23.7–39.3)^a^ Critical: 25.4 (16.3–30.6)^a^ Non-critical: 35.7 (28.1–41.3)^a^	NR

*Values are mean ± SD unless otherwise specified*.

a *Median (IQR)*.

b *Median (min–max)*.

* *Unpublished data*.

# *Value are in cm^1^*.

*NR, Not reported; SMI, Skeletal muscle index; HU, Hounsfield units*.

#### Muscle Density

**Figure 4:**
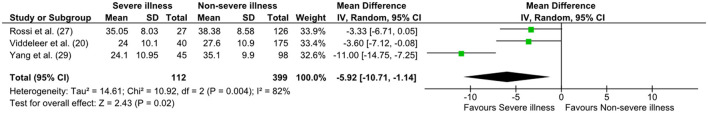
The forest plot of muscle density status and survival rate. SD, standard deviation; CI, confidence intervals; HU, Hounsfield units.

A total of three articles reported muscle density, Rossi et al. (27); Viddeleer et al. (20); Yang et al. (29), providing CT analysis of 511 patients. The pooled effect size of this subgroup analysis showed that low muscle density was associated with high mortality [MD = 5.92; 95% CI: −10.71, −1.14; *Z* = 2.43; *p* = 0.02 (Figure 4)]. and heterogeneity was significant (*I*^2^ = 82%, *p* = 0.02). Two studies significantly contributed to this result (20, 29). In the study of Rossi et al. (27), participants with lower muscle density (assessed by CT at the L3–L4 level) values showed shorter survival within 28 days from ICU admission, as compared to subjects in the highest muscle density specter [Hazard ratio (HR) 3.27, 95% CI: 1.18, −4.61]. However, in this meta-analysis, muscle density from the study of Rossi et al. (27) presented no effect in the subgroup overall analysis. In another study, Yang et al. (29), examining critically ill and non-critically ill groups (median value of muscle density 25.4 HU and 35.7 HU, respectively), found the difference to be statistically significant (*p* < 0.001). Viddeleer et al. (20) reported no differences for muscle density at the level of the 12th thoracic vertebra between survivors and non-survivors (*p* = 0.067). The overall effect analysis has shown that Yang et al. (29) and Viddeleer et al. (20) have similar weight (33.4 and 33.9%, respectively) in our analysis.

#### Handgrip Strength

**Figure 5:**
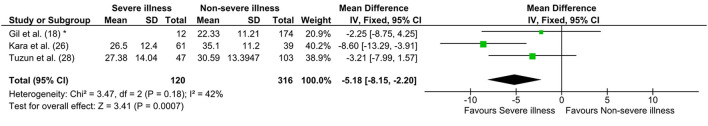
The forest plot of hand grip status and survival rate. *Unpublished data. SD, standard deviation; CI, confidence intervals.

Three articles assessed grip strength using a dynamometer, with heterogeneity of 42%. Lower handgrip strength was associated with COVID-19 severity [MD = 5.18; 95% CI: −8.15, −2.20; *Z* = 3.41; *p* = 0.0007 (Figure 5)]. Gil et al. (18) reported that patients who survived COVID-19 had a median and interquartile range handgrip strength of 22 (15–30). For this meta-analysis, the median and interquartile range of grip strength employed were those of the patients who did not survive (data not shown, kindly provided by the authors). Kara et al. (26) showed that patients with severe disease presented lower grip strength (26.5 kg/f ± 12.4) compared to patients with moderate (34.7 kg/f ± 11.1) and mild disease (35.1 kg/f ± 11.2). Tuzun et al. (28) reported handgrip measurement by disease severity and gender. Female patients with non-severe disease (23.37, CI 21.45, 25.48; showed higher handgrip strength than female patients with the severe form of the disease (18.26, CI 15.19, 21.68). Male patients with non-severe disease (37.67, CI 33.42, 41.39) did not differ from those with severe disease (35.40, CI 29.56, 40.89). Overall, COVID-19 severity was associated with low muscle quality and function (MD = −3.75; 95% CI: −6.20, −1.29; Z = 2.99; *p* = 0.003, *I*^2^ = 71%) (28).

The overall effect of this meta-analysis indicated that lower muscle quality and function were related to disease severity, as showed in Figures 4, 5 by the black diamond positioned at the left side of vertical line of the absence effect.

## Discussion

Assessment of muscle mass can be carried by out employing different tools, with variable reliability. Handgrip strength evaluation may pose a difficult task in ICU, hence the adoption of other means to provide insight on muscle quality including CT, which is often available for COVID-19 patients, and can be an important predictor of the disease severity (31).

Cross-sectional CT images at the L3 level strongly correlate with body adipose tissue, appendicular skeletal muscle mass, and fat-free body mass content (32, 33). CT provides muscle and fat tissue images through x-ray attenuation rates and allows the determination of intramuscular lipid content, which is associated with lower muscle quality, and consequently, with lower muscle strength (34). Nonetheless, CT image analysis may be have limitations due to the difficulty distinguishing between intra-myocellular fat and inter-muscular fat (35). The muscles evaluated at this level are the *rectus abdominis*, internal oblique, external, transverse*, psoas major, quadratus lumborum*, and *erector spinae* (32, 33).

The most commonly used CT image level to analyze body composition is L3/L4, although images at the thoracic vertebra T12 level can be also adopted due to their good correlation with whole-body composition, and this type of image (36), is frequently available for COVID-19 patients. At the T12 level, external and internal oblique*, rectus abdominis, erector spinae, latissimus dorsi*, and external and internal intercostal muscles are evaluated (36). Given the above, skeletal muscle index data from two studies that evaluated muscle mass at T12 and one study measuring at L3 were combined to investigate SMI association with COVID-19 severity. The results of this meta-analysis showed no association between SMI and COVID-19 severity.

This meta-analysis shows that rather than mass, skeletal muscle quality is associated with COVID-19 severity. This goes in agreement with recent findings implying that muscle quality is a more relevant factor for disease prognosis than actual muscle quantity, as also found for cancer, major surgery„ aging, and liver disease (37–39). The main result of the study demonstrates that muscle density, not quantity, confers an important prognostic value for the severity of COVID-19 in infected patients.

Skeletal muscle density can be determined, among other factors, by the amount of intramuscular fat. In fact, recently published studies have shown that the amount of intramuscular fat interferes with the prognosis of COVID-19 (20, 29, 40).

Yang et al. (29) reported that critically ill COVID-19 patients showed lower muscle density. These patients also show higher greater intramuscular fat (IMF) deposition, or higher visceral adiposity and present a higher risk for the requirement of mechanical ventilation. Additionally, the group that presented a higher IMF was also of more advanced age and showed higher risk of death. Therefore, body composition and muscle quality are important parameters to consider in patients with COVID-19 (29). Another study reported no association of muscle density with worsened patient outcome (20), yet a larger area of intramuscular adipose tissue at the level of T12 was a risk factor decreasing survival in COVID-19 (20).

In addition to muscle quality, the results of this meta-analysis demonstrate that muscle function also has prognostic value in patients with COVID-19. Gil et al. (18), investigated the relationship between muscle strength (by handgrip) and muscle mass (by ultrasound) as predictors of length of stay (LOS) in patients with moderate to severe COVID-19. Although the sample was heterogeneous and the patients presented different comorbidities, being also under different medication regimens, and presented heterogeneous adverse clinical manifestations, the study succeeded in demonstrating that muscle strength and mass assessed upon hospital admission are robust predictors of LOS in these patient population. Furthermore, the same study emphasized that these data can help predict the risk of illness severity.

Tuzun et al. (28) discussed the limitations of their study, such as the need for more samples per group and as well more heterogeneous samples to analyze. However, the literature also shows that “low” grip strength values determine specific clinical attention and should be considered a resource in rehabilitation strategies for patients with COVID-19 (28). Kara et al. (26) showed that lower handgrip strength as well as age, obesity, chronic obstructive pulmonary disease and C-reactive protein levels were all associated with severity of COVID-19. All things considered, these findings highlight the importance of muscle-related parameters assessment for establishing the prognosis of the disease.

Decreased muscle strength (dynapenia) may be expected in many patients. The causes include age, malnutrition or illness (41). Additionally, the decrease in food consumption or difficulty absorbing nutrients, common in institutionalized patients, results in depletion of total body protein, whose most significant reserve is in muscles (41, 42). Dynapenia is associated with unfavorable disease outcomes, such as extended hospital stay, increased function limitations, poor health-related quality of life and increased mortality (12, 41, 42).

The impact of muscle depletion in institutionalized individuals with different clinical conditions has been broadly studied (43–46). Quantitative and qualitative muscle mass deterioration, is relatively frequent in patients during hospitalization due to several factors including systemic inflammation, presence of comorbidities, requirement for mechanical ventilation, multiple organ dysfunction, and immobility for an extended period (43–46). In critically ill patients, myopenia is correlated with extended mechanical ventilation, prolonged ICU stays, and poor survival, among other complications (43). The stress response to trauma and immobility causes negative protein balance and also, resistance to anabolic signaling, leading to proteolysis and loss of muscle mass, which are characteristics of sarcopenia (47). Up to 63% of individuals admitted to the ICU on ventilatory support present low muscle mass, notably the elderly (47).

In line with this, Loosen et al. (48) have demonstrated, by using a biometric software, the role of sarcopenia and myosteatosis as prognostic factors in critical ICU patients. Through L3-SMI and mean skeletal muscle attenuation (MMA) assessment, the authors showed that low MMA and low L3-SMI together are able to predict the overall survival in critical ill patients. The combination of L3-SMI and MMA was superior to either marker alone, highlighting that myosteatosis and sarcopenia might reflect specific aspects within different diseases (48).

Systemic inflammation is another aspect with a high potential to influence body composition and muscle function. Cole et al. (49) showed that this process contributes to the stimulus of proteolysis, and to upregulation the proteasome pathway, which can subsequently increase myostatin and activin A release, causing the suppression of muscle protein synthesis (49–51). Therefore, in addition to the amount of muscle mass, it is essential to analyze muscle quality loss and the respective presence of IMF, which is related to decreased muscle strength, inflammation, and insulin resistance (52). However, it is not the aim of the present meta-analysis to address the impact of chronic or acute inflammation on muscle mass and quality loss and respective association with COVID-19 outcome.

For a comprehensive discussion of this topic, we suggest the recent reviews by Tuttle et al. (53).

In a recent systematic review and meta-analysis, systemic inflammation, characterized by increased CRP, IL-6 and TNF-alpha levels, was related to lower muscle strength and lower muscle mass (53). Another meta-analysis showed that sarcopenia was associated with systemic inflammation, mainly characterized by high levels of CRP (6). The skeletal muscle secretes hundreds of myokines that modulate insulin sensitivity, inflammation, immune function, lipid oxidation, and body metabolism (43). Furthermore, skeletal muscle quality contributes to the individual's physical strength and ability to carry out daily activities, and the loss of the quality of this body compartment has a marked adverse impact on the clinical outcome and survival in various diseases, such as obesity, cancer, diabetes, obstructive pulmonary disease, muscle diseases, liver disease, etc. (39, 43).

### Strengths and Limitations

The studies presented an extensive age range, which is representative of SARS-CoV-2 infection. However, this variability creates a factor of heterogeneity that may influence the results. Skeletal muscle mass, quality and function are strongly affected by age and sex, and this study could not stratify the patients taking these confounding factors into account. Although authors reported these data, we did not evaluate the frequency and percentage of comorbidities in the studies included. That represents another limitation that could increase sample heterogeneity. Nevertheless, all the studies presented a low risk of bias and high quality of study design. Moreover, muscle density analysis showed high heterogeneity. More studies evaluating muscle density in patients with COVID-19 are necessary to confirm the findings reported here.

## Conclusion

This meta-analysis showed that low muscle quality and function, rather than muscle quantity/mass, are associated with COVID-19 disease severity. Therefore, muscle function should be included as a clinical predictor in the evaluation of these patients. In addition, mechanistic studies are necessary to understand better the influence of muscle quality in clinical COVID-19 prognostic.

## Data Availability Statement

The original contributions presented in the study are included in the article/Supplementary Material, further inquiries can be directed to the corresponding author.

## Author Contributions

AB, FP, GSa, GSi, JF, MA, and RG: were involved in the study design, data collection, drafting the manuscript, contributed to data interpretation, and article writing. FP: collation of results and analysis. MS: critically review, data interpretation, and article writing. All the authors read and approved the final manuscript.

## Funding

We acknowledge the São Paulo Research Foundation (FAPESP Grants 20/07765-6 and 12/50079-0 to MS) and the Fundação Faculdade de Medicina for financial support.

## Conflict of Interest

The authors declare that the research was conducted in the absence of any commercial or financial relationships that could be construed as a potential conflict of interest.

## Publisher's Note

All claims expressed in this article are solely those of the authors and do not necessarily represent those of their affiliated organizations, or those of the publisher, the editors and the reviewers. Any product that may be evaluated in this article, or claim that may be made by its manufacturer, is not guaranteed or endorsed by the publisher.
